# Cellular senescence in musculoskeletal homeostasis, diseases, and regeneration

**DOI:** 10.1038/s41413-021-00164-y

**Published:** 2021-09-10

**Authors:** Mei Wan, Elise F. Gray-Gaillard, Jennifer H. Elisseeff

**Affiliations:** 1 Department of Orthopaedic Surgery, The Johns Hopkins University School of Medicine, Baltimore, MD USA; 2 Translational Tissue Engineering Center, Wilmer Eye Institute and the Department of Biomedical Engineering, The Johns Hopkins University, Baltimore, MD USA

**Keywords:** Bone, Osteoporosis

## Abstract

Emerging insights into cellular senescence highlight the relevance of senescence in musculoskeletal disorders, which represent the leading global cause of disability. Cellular senescence was initially described by Hayflick et al. in 1961 as an irreversible nondividing state in in vitro cell culture studies. We now know that cellular senescence can occur in vivo in response to various stressors as a heterogeneous and tissue-specific cell state with a secretome phenotype acquired after the initial growth arrest. In the past two decades, compelling evidence from preclinical models and human data show an accumulation of senescent cells in many components of the musculoskeletal system. Cellular senescence is therefore a defining feature of age-related musculoskeletal disorders, and targeted elimination of these cells has emerged recently as a promising therapeutic approach to ameliorate tissue damage and promote repair and regeneration of the skeleton and skeletal muscles. In this review, we summarize evidence of the role of senescent cells in the maintenance of bone homeostasis during childhood and their contribution to the pathogenesis of chronic musculoskeletal disorders, including osteoporosis, osteoarthritis, and sarcopenia. We highlight the diversity of the senescent cells in the microenvironment of bone, joint, and skeletal muscle tissue, as well as the mechanisms by which these senescent cells are involved in musculoskeletal diseases. In addition, we discuss how identifying and targeting senescent cells might positively affect pathologic progression and musculoskeletal system regeneration.

## Introduction

The musculoskeletal system (MSK), including bone, cartilaginous tissues, skeletal muscle, tendon, ligament, intervertebral disc, and others, is the structural framework for the body and enables movement. Bones are held in position by ligaments. Skeletal muscles power the movement of bones. Muscles and bones are connected through tendons. These tissues together form the articulating joints. In addition to providing physical support, MSK tissues have endocrine functions and communicate with other tissues to affect organism homeostasis and overall physiological health. The skeleton is a highly dynamic structure that changes in shape and composition throughout life. Skeletal muscle fiber also has high remodeling plasticity on demand. Not surprisingly, bone, cartilage, and skeletal muscle cell homeostasis is tightly controlled, as is the maintenance of tissue structure and mass. Aging, characterized by a gradual functional decline, is the greatest risk factor for many chronic musculoskeletal conditions, such as osteoporosis, osteoarthritis, and sarcopenia. As common musculoskeletal complications, these three disorders share genetic, endocrine, and mechanical risk factors, and are also closely connected both mechanically and metabolically. These age-associated musculoskeletal conditions leads to MSK structural degeneration, mechanical pain, decreased mobility, and limited function.^1,2^ Particularly, among patients aged 65 years or older, chronic skeletal diseases, such as osteoporosis and associated bone fracture and osteoarthritis, are the most prevalent conditions leading to frailty and a decline in mobility. An estimated 10 million Americans have osteoporosis, and another 43 million have low bone density. Osteoporosis will be responsible for more than $25 billion in annual health care spending by 2025 in the United States.^3^ Osteoarthritis is the most prevalent chronic joint disease affecting the knees, hands, hips, and spine, and is the fourth most common cause of hospitalization among adults in the United States, resulting in enormous health care costs.^4^ Sarcopenia is present in an estimated 5%–13% of individuals older than 60 and 50% of persons older than 80,^5^ and can cause severe adverse clinical outcomes.^6^ Despite substantial social demand, no medications for osteoarthritis or sarcopenia disease treatment have been approved by the US Food and Drug Administration. Related to their structural function, MSK tissues are susceptible to trauma and injury, and aging also adversely affects the tissue regenerative capacity, resulting in delayed tissue repair, disability, and pain. In the past few years, the MSK research community has been considerably interested in cellular senescence, which represents a series of diverse, dynamic, and heterogeneous cellular states with the senescence‐associated secretory phenotype (SASP). In this review, we provide a comprehensive summary of cellular senescence known to be involved in MSK homeostasis, disease, and regeneration. As senescent cells (SnCs) appear to have dual roles in physiologic tissue homeostasis/repair as well as pathologic responses, this review highlights the functional heterogeneity and the molecular pathways responsible for senescence induction in distinct physiological settings.

## Pathways triggering cellular senescence and the SASP

**Fig. 1:**
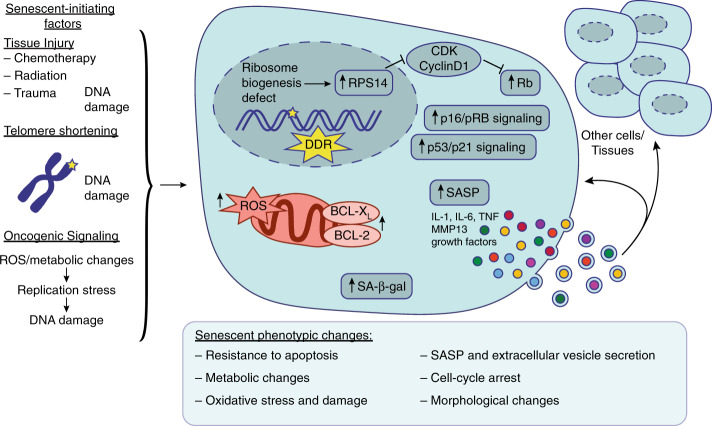
Overview of senescence. Several factors can induce senescence in different tissues, such as tissue injury, telomere shortening, and oncogenic signaling that all lead to DNA damage, the DNA damage response (DDR), and consequent cell cycle arrest by activation of p16/pRB signaling and/or p53/p21 signaling. Nucleolar stress and ribosome biogenesis defects can also induce RPS14 accumulation in the nucleus and activates Rb by inhibiting the CDK4/cyclin D1 complex, leading to cell cycle arrest. Senescent cells also exhibit increased senescence-associated β-galactosidase (SA-β-gal) production, reactive oxygen species (ROS) accumulation, and anti-apoptotic factors such as BCL-X_L_ and BCL-2. Senescent cells exhibit several phenotypic changes such as a resistance to apoptosis, oxidative stress and damage, metabolic changes, morphological changes, cell cycle arrest, and extracellular vesicle secretion containing SASP factors such as IL-1, IL-6, TNF, MMP13, and various growth factors. This SASP can either feedback in an autocrine manner to the senescent cell or in a paracrine manner influence and promote senescence and inflammation in the surrounding cells and tissues

Cellular senescence, initially described by Hayflick et al. in 1961 as limited replicative potential of normal cultured human fibroblasts,^7,8^ was thought to be an in vitro phenomenon. Starting at the beginning of the twenty-first century, work by multiple groups showed that these SnCs are also abundant in vivo. Based on the consensus reached by the International Cell Senescence Association, cellular senescence is now viewed as a cell state triggered by stressful insults and certain physiologic processes, characterized by a prolonged—and generally irreversible—cell cycle arrest with secretory features, macromolecular damage, and altered metabolism (Fig. 1).^9–14^ The SnCs can affect their local tissue environments through the SASP. Importantly, removal of SnCs in adult mice led to major improvements in health span and lifespan. The recent generation of senescence reporter/ablation mouse models and the development of senotherapies have advanced our knowledge of the phenotypic features and pathogenesis of the SnCs, as well as their contributions to physiologic and pathologic processes.

### SnC cycle arrest and related senescence biomarkers

Irreversible cell cycle arrest is a defining characteristic of cellular senescence despite its many facets. Earlier studies of the mechanisms of the replicative senescence that causes stable cell cycle arrest found that replicative senescence in human cells is provoked by telomere erosion after extensive serial passaging.^15–17^ Cells can also undergo senescence independent of telomere shortening, and senescence can be induced when cells are exposed to genotoxic agents, such as reactive oxygen species, deoxyribonucleic acid (DNA)-damaging agents, hypoxia, mitochondria dysfunction, certain activated oncogenes, and epigenetic alteration.^18–21^ Two main tumor suppressor-mediated signaling pathways, p53/p21^CIP1^ and p16^INK4a^/pRB, are responsible for the growth arrest of SnCs.^22–25^ The p53/p21^CIP1^ pathway is activated downstream of the DNA damage response from DNA double-strand breaks or uncapped telomeres. p16^INK4a^/pRB signaling initiates and maintains permanent cell cycle arrest. The *INK4A/ARF* locus is normally epigenetically silenced by polycomb repressive complexes. Disrupting the activities of polycomb repressive complexes derepresses p16^INK4a^ and induces senescence.^26–28^ p16^INK4A^ activation inhibits CDK4 and CDK6 activity, which leads to RB hypophosphorylation, blockade of S-phase entry, and cell cycle arrest.^29^ Ribosome biogenesis defect is another feature of SnCs and also an important contributor to cell cycle arrest. The involvement of ribosome biogenesis perturbation in cellular senescence was first reported in a study showing that excessive rRNA transcription in oncogene-induced senescence or inhibition of rRNA processing in replicative senescence generates nucleolar stress, which drives cell cycle arrest through p53.^30^ The mechanisms of how ribosome biogenesis defect drives cellular senescence have been further revealed recently. Specifically, accumulation of the ribosomal 40S subunit protein RPS14 is a key contributor to senescence in response to multiple stimuli, and the capacity of RPS14 to trigger cellular senescence is CDK4/Rb-dependent and p53-independent.^31^ RPL22/eL22, another ribosomal protein, is sufficient to induce a cellular senescent phenotype by inhibiting CDK4-Cyclin D1 to decrease RB phosphorylation.^32^ Moreover, inhibition of the reaction catalyzed by LSG1, a GTPase involved in the biogenesis of the 60S ribosomal subunit, leads to a robust induction of cellular senescence that is associated with perturbation of endoplasmic reticulum homeostasis.^33^ We recently found that reduced/loss of expression of a ribonuclease angiogenin (ANG) in osteoclasts in long bone metaphysis leads to cellular senescence of neighboring vascular cells by inducing ribosomal biogenesis deficit.^34^ This finding provides more evidence on the role of ribosome biogenesis damage as a trigger of cellular senescence.

Despite different pathways identified in mediating SnC cycle arrest, no consensus exists regarding the biomarkers of the SnCs.^35^ Generally, none of the commonly used senescence-associated markers are specific or universal for all cell types, making identification of SnCs challenging. For example, senescence-associated beta-galactosidase was the first documented biomarker of senescence;^36^ however, it can also be detected in non-senescent macrophages,^37^ osteoclasts,^38^ and some other cell types, especially those in stressed states.^39^ p16^INK4a^ and p21^WAF1/Cip1^ are also commonly used markers of cellular senescence, but they are not expressed in all SnCs,^35^ and their expressions depend on the specific type of senescence program.^40^ γ-H2AX and 53BP1, DNA damage response proteins, are also often used as SnC markers, but they also appear in transient DNA-damaged non-SnCs.^41^ Quantification of senescence-associated distension of satellites (SADS) (i.e., unraveled centromeres)^42–44^ and telomere-associated foci (i.e., sites of DNA damage within telomeres)^45^ have also been used to evaluate cellular senescence. Ribosomal protein RPL29 accumulates in nucleoli in response to cellular senescence stimuli, and therefore can serve as a new biomarker for cellular senescence.^31^ Reduced or loss of expression of cell-proliferating proteins Ki67^46^ and nestin,^47–49^ and loss of Lamin B1^50^ and HMGB1^51^ in the nucleus are also important features of SnCs. However, Ki67 and nestin loss are also observed in quiescent or terminally differentiated cells. A recent study reported that nuclear nestin deficiency drives tumor senescence via Lamin A/C-dependent nuclear deformation, providing a mechanism by which nestin loss may be involved in cellular senescence.^52^ Because none of the genes described above appear to be expressed uniquely by SnCs, the levels of multiple transcripts should be measured at the same time and in the same sample to avoid false positives. Importantly, it has been shown, by comparing whole-transcriptome datasets from different types of SnCs, that there is a “core” senescence signature—a set of genes that are commonly differentially expressed in the SnCs.^53,54^ A newly developed two-phase algorithmic assessment for quantification of various senescence-associated parameters in the same specimen^55^ will be helpful to overcome the limitation of measuring individual SnC markers.

### SASP

In addition to stable cell cycle arrest, the SASP or senescence-messaging secretome is another key feature of SnCs that distinguishes the cells from other cell cycle-arrested cells. SnCs secrete hundreds of factors that include proinflammatory cytokines, chemokines, growth factors, and proteases.^9,56–59^ A recent study reported that oxylipins, a class of biologically active lipids that arise from the oxygenation of polyunsaturated fatty acids, are a component of the SASP.^60^ Not only do SnCs with SASP induce autocrine reinforcement, but they also communicate with neighboring cells in a paracrine manner to propagate the stress response in the tissue microenvironment.^56,61,62^ Of note, the paracrine effects of the SASP can be beneficial or detrimental depending on the tissue context.^63^ On the one hand, the SASP prevents cancer progression by inducing paracrine senescence in neighboring cells,^61,62^ promoting embryogenesis, tissue repair, and regeneration,^58,64,65^ and activating the immune system, which facilitates clearance of damaged cells.^66,67^ On the other hand, the SASP promotes tumorigenic processes such as angiogenesis and invasion^56,68^ and induces chronic inflammation that drives aged-related abnormalities in various tissues.^69,70^ The SASP factors produced by SnCs are highly cell specific and vary substantially even in the same type of cells, depending on the duration of senescence and origin of the pro-senescence stimulus.^53^ Through mass spectrometry, a core set of SASP proteins that are commonly produced by different types of SnCs were identified,^54^ which is helpful for the prediction of senescence-associated functions. It is important to note that despite the existence of certain core proteins, the expression and secretion of most SASP factors remain variable and context dependent. SASP induction and cell proliferative arrest appear to be regulated through separate signaling pathways because SASP is not induced by the expression of master cell cycle arrest regulatory genes p16^Ink4a^ or p21^Waf1/Cip1^.^57,71^ Reportedly, transcriptional activation, chromatin remodeling, damaged DNA sensing, and extracellular vesicles, such as exosomes, may cause the activation of the SASP gene and promote the development of SASP. This aspect of SnCs has been summarized in several recent review articles.^72–76^ Although multiple pathways have been reported for SASP initiation and regulation of SnCs, the precise mechanisms regulating SASP induction are far from understood. Further defining the senescent secretome and its heterogeneity in function in various biological contexts and better understanding the temporospatial regulation of the SASP will help identify more biomarkers of SnCs, enabling the development of specific senescence modulatory therapies.

## Cellular senescence in bone homeostasis, disease, and repair

### Childhood bone homeostasis and diseases

**Fig. 2:**
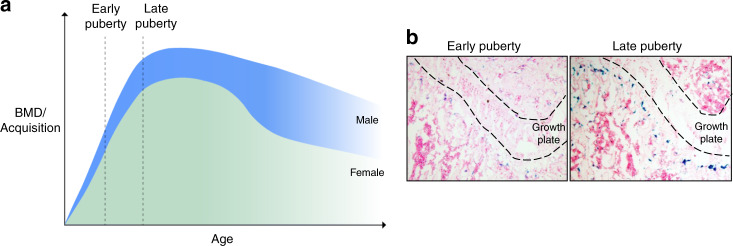
Cell senescence in long bone during late puberty is a programmed physiological process. **a** Changes in bone mineral density (BMD)/bone acquisition across the lifespan. BMD/acquisition increases rapidly during childhood and early puberty periods, becomes slow during late puberty, and reaches a plateau in young adulthood. **b** SA-βGal staining images show increased senescent cells in primary spongiosa in mice during late puberty (8 weeks old) relative to early puberty (3 weeks old)

Human bones change throughout the lifespan. Bone growth is characterized by a sharp increase during early puberty and deceleration and eventual cessation during late puberty. As growth in length accelerates, bone mass accrual also increases markedly during childhood and adolescence but gradually slows and eventually ceases during late puberty and young adulthood^77,78^ (Fig. 2a). At the cellular level, the growth plate at the ends of long bones and the adjacent primary spongiosa undergo substantial changes during late puberty to adapt to the much slower bone growth/accrual during this period. Vascular endothelial cells that form invaded blood vessels and mesenchymal stem/progenitor cells that replenish bone-forming osteoblasts are highly proliferative during the rapid bone-growth period, but these cells likely stop proliferating or are replaced by other cell types when bone growth stops. We uncovered a bone-growth cessation-associated cellular senescence at this particular time of life.^47^ We found that stem/progenitor cells in the metaphysis of long bone are highly proliferative during early puberty but undergo progressive cell senescence during late puberty (Fig. 2b). Cellular senescence was defined by the presence of SA-βGal and p16INK4a and decreased expression of Ki67 and nestin. We also found that the senescence process is controlled by an epigenetic mechanism.^47^ Ezh2-H3K27me3 maintains the self-renewal and proliferative capacity of cells in primary spongiosa of fast-growing bones, whereas loss of Ezh2-H3K27me3 during late puberty leads to cell senescence. Bone cell senescence in late puberty should be a normal physiological event because it is restricted to a particular region of the long bone, follows a specific time course, and is programmed by a conserved mechanism. This senescence program during late puberty somewhat resembles the senescence process that has been recognized during embryogenesis, in which the SnCs are thought to play a beneficial role in establishing the initial formation of tissues/organs.^64^ Regarding the skeletal system, SnCs were found in embryonic limbs at the apical ectodermal ridge, which is the main region coordinating limb outgrowth and patterning. Loss of senescence by p21 knockout results in impaired pattern formation and proliferation of the mesenchyme cells,^65^ reflecting the beneficial role of senescence in embryonic skeletal development.^64,65^ Importantly, embryogenesis-associated senescence is also time and location restricted.^64,79^ It seems that, during development, SnCs can be automatically eliminated by apoptosis or immune cells.^64,65,80^ Therefore, the remodeling of developing structures can be accomplished by promoting immune-mediated cell clearance of particular cell populations or by modifying the tissue microenvironment.^81^ It is of interest to test whether immune cells are also involved in clearing the SnCs in bone metaphysis during late puberty. In future studies, it will also be important to determine whether SnCs are increased in the cortical bone or bone tissue in other locations (e.g., lumbar vertebrae, skull) during the postnatal period, when the rates of bone growth and mineral acquisition slow. Further uncovering of the detailed mechanisms that control skeletal (bone and cartilage) cell senescence during embryonic development and childhood growth will provide exciting new opportunities to understand the pathogenic mechanisms associated with congenital and childhood skeletal abnormalities.

**Fig. 3:**
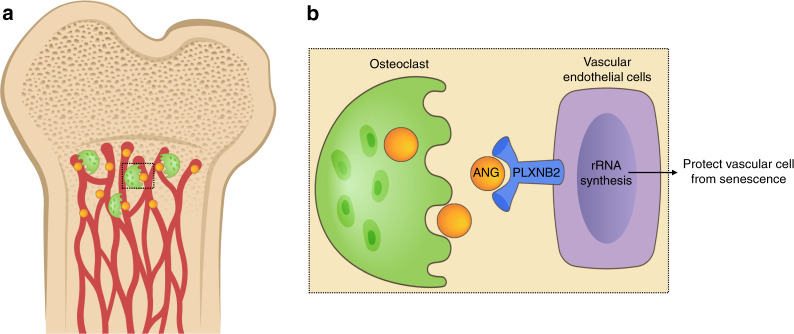
Osteoclasts protect bone blood vessels against senescence through the ANG/PLXNB2 axis. **a** ANG secreted from metaphyseal osteoclasts maintains blood vessel homeostasis in growing long bone through an ANG/PLXNB2-rRNA transcription signaling. **b** Enlarged image for the black square in (**a**)

In children with genetic skeletal disorders or chronic disease, bone growth and mineral accrual are often compromised, leading to osteoporosis and high rates of bone fracture. During childhood, fractures are common, with an annual fracture incidence of 205 per 10 000 person-years in those younger than 16 years, with the highest rate during the rapid growth spurts of puberty.^82,83^ Up to 25% of children with a fracture history experience more than one fracture, often representing primary or secondary bone loss or osteoporosis.^82^ Moreover, bone mineral accrual during childhood and adolescence influences long-term bone health. Epidemiologic studies suggest that 60% of the risk for osteoporosis later in life can be explained by the bone mineral acquired by childhood and early adulthood. Pediatric osteoporosis is often caused by an underlying medical condition (i.e., secondary osteoporosis) or a genetic disorder. Glucocorticoid-induced osteoporosis is the most common form of secondary pediatric osteoporosis. Long-term glucocorticoid therapy has been used widely in the treatment of chronic inflammatory childhood illnesses. Although the use of glucocorticoids has led to improved outcomes and survival rates, it has major adverse effects on bone. Epidemiologic studies have shown a prevalence of up to 34% for vertebral fractures in children and youth who have undergone long-term glucocorticoid therapy.^84^ We recently found that glucocorticoid treatment in young mice induces vascular endothelial cell senescence in metaphysis of long bone, and that inhibition of endothelial cell senescence improves glucocorticoid-impaired bone angiogenesis with coupled osteogenesis.^34^ We identified ANG, a ribonuclease that is secreted by osteoclasts and that has pro-angiogenic activity, as a key factor for protecting the neighboring vascular cells against senescence. We found evidence that ANG secreted from metaphyseal osteoclasts is essential for maintaining the closely associated blood vessels in growing long bone through an ANG/PLXNB2-rRNA transcription signaling pathway (Fig. 3). Glucocorticoid treatment inhibits ANG production through suppression of osteoclast formation in metaphysis, leading to blood vessel cell senescence and impairment of angiogenesis with coupled osteogenesis. Our results reveal cellular senescence as a new line of mechanisms for the deleterious effects of glucocorticoids on the growing skeleton and suggest that the ANG/PLXNB2 axis functions as a molecular basis for the osteoclast-vascular interplay in maintaining bone blood vessels from senescence. An important future question is whether the decline or loss of ANG/PLXNB2 signaling in adult bone is also a key mechanism leading to cellular senescence during aging or under disease conditions.

### Osteoporosis with aging

Bone aging is a normal process caused by the disrupted balance between bone resorption and formation as a result of the changes in osteoblast and osteoclast activity, leading to bone loss. In fact, peak bone mass (PBM) occurs during the third decade of life, after which bone mass plateaus and then declines. Men and women have different rates of bone loss after PBM. Markedly accelerated bone loss in women occurs during perimenopause and the early postmenopausal period. In men, bone loss with aging is less profound but is maintained at a persistent lower rate.^2,85,86^ In humans and mice, bone loss differs between the trabecular and cortical compartments with age. Trabecular bone aging is associated with reduced trabecular number, increased spacing, and decreased thickness.^87,88^ In cortical bone, endocortical resorption and periosteal bone formation often occur during aging, leading to cortical thinning with marrow cavity expansion and increased cortical porosity.^2,89,90^ In addition to the reduction in bone quantity, bone quality and strength also diminish during aging, leading to increased fracture risk, especially from falls. Bone aging is mediated by complex interactions of cells at multiple levels with the combination of intrinsic changes and extrinsic factors. Intrinsic changes include alterations in hormone status, cell components, and gene expression, as well as local microenvironmental changes caused by the altered production of growth factors, cytokines, and inflammatory factors. Extrinsic factors include nutrition and comorbid medical conditions. At the molecular level, it has been proposed that genomic instability, epigenetic alterations, telomere attrition, loss of proteostasis, mitochondrial dysfunction, deregulated nutrient sensing, and cellular senescence gene activation may contribute to age-related dysfunction in bone.^91–93^

In the past few years, there is growing evidence to suggest that cellular senescence is a major player in the pathogenesis of osteoporosis, a prevalent age-related bone disorder characterized by deterioration of bone microarchitecture and low bone mineral density (BMD). SnC accumulation has been observed in bone/bone marrow, as shown by increased SA-βGal, SADS, telomere dysfunction-induced foci, p16INK4a, or p21CIP1 expression in many types of cells in aged mice, including bone marrow myeloid cells, T cells, B cells, MSCs, osteoprogenitors, osteoblasts, and osteocytes.^42,89,94^ These SnCs also acquire SASP and secrete various factors to affect their microenvironment, leading to the loss of bone mass and architecture. SASP secretion of bone marrow MSCs and osteoblastic cells mediates enhanced osteoclastogenesis and the development of osteoporosis.^42,95^ The study of senescence and the SASP of osteocytes, the longest-living bone cells embedded in the bone matrix, has drawn much attention. Senescent osteocyte accumulation has been found in the aged bone environment of mice and humans,^42,89^ and induction of osteocyte senescence is sufficient to stimulate receptor activator of nuclear factor kappa-B ligand production, leading to increased osteoclast formation and activity.^96^ The presence of SnCs and the corresponding SASP in osteoporotic bone has also been confirmed in elderly people.^42^ Importantly, the genetic elimination of the SnCs using INK-ATTAC mice or administration of senolytic drugs was able to inhibit production of SASP in the bone marrow microenvironment, leading to increased bone mass and microarchitecture in aged osteoporotic mice.^97^ Therefore, targeting cellular senescence may represent a new therapeutic strategy to prevent or treat osteoporosis in the elderly. However, activation of *p16-3MR* transgene failed to eliminate senescent osteocytes and alleviate age-associated bone loss,^98^ indicating that genetic approaches used for the elimination of p16‐expressing cells are generally tissue selective. Another interesting question is the role of cellular senescence in accelerated skeletal aging in response to sex steroid deficiency. Estrogen has a crucial effect on bone metabolism, and its decline is causal in the pathogenesis of osteoporosis. In one report, ovariectomy led to the generation of premature T-cell senescence in mice, and estrogen treatment reversed the phenotype.^99^ However, recent work by Farr et al.^100^ using multiple approaches in mice and humans suggests no evidence of changes in senescence biomarkers or SASP factors in mouse or human bone in response to estrogen deficiency. Moreover, elimination of SnCs during young adulthood does not restore the bone loss caused by estrogen deficiency. Therefore, it is likely that estrogen deficiency-induced bone loss occurs independently of cellular senescence.

### Osteoporosis in diabetes mellitus

Osteoporosis, bone fractures, and bone-related disorders are more common in people with diabetes mellitus (DM) than in the general population. DM can cause osteopenia and osteoporosis and aggravates both conditions. Pathologic bone changes differ between those with type 1 DM (T1DM) and type 2 DM (T2DM). Patients with T1DM often develop osteoporosis with reduced BMD at an early age, whereas bone in T2DM exhibits normal or high BMD. However, patients with T2DM have a higher rate of fractures, mainly because of compromised bone quality and microarchitecture.^101,102^ Eckhardt et al.^103^ examined cellular senescence in a T2DM mouse model, in which accelerated accumulation of senescent osteocytes with SASP was detected in bone while bone microarchitecture and biomechanical strength were compromised. The finding suggests that the SnCs and corresponding SASP are likely involved in skeletal fragility in T2DM. It remains unclear whether cellular senescence and SASP are involved in the pathogenesis of T1DM-related bone loss. Future studies are needed to determine whether other cell types in the bone/bone marrow microenvironment also undergo cellular senescence with DM and whether they are factors in the disease pathogenesis. SnCs accumulate in adipose tissue of humans and mice with T2DM, obesity, and age-related metabolic dysfunction.^104^ Given that bone mass/quality is negatively correlated with many of these metabolic disorders, and more fat cells are present in the bone marrow in these conditions, it has long been proposed that increased marrow adipocyte differentiation from mesenchymal stem/progenitor cells accounts for age- and DM-associated bone loss. However, a recent study showed that increased marrow adipogenesis did not contribute to age‐ and DM-associated appendicular bone loss in female mice,^105^ and provides a compelling argument against the above hypothesis. Additional studies are still needed to clarify whether bone marrow adipocytes have a senescence phenotype and secrete SASP factors during DM to regulate the function of surrounding bone cells.

## Cellular senescence in osteoarthritis development

### Cellular senescence in the pathogenesis of osteoarthritis

The articular cartilage in human joints is subject to extreme biologic and biomechanical demands in allowing movement and function. The term “articular cartilage” typically refers to hyaline cartilage, which must be maintained within a narrow range of biochemical composition and morphologic architecture to maintain its integrity, and the intrinsic healing potential of articular cartilage is very limited. Therefore, any insult, such as injury, aging, or metabolic dysregulation, can initiate a cascade of events that lead to degradation and erosion of articular cartilage and the development of osteoarthritis. Osteoarthritis is a common chronic musculoskeletal disease and a leading cause of disability with no effective disease-modified therapy.^41,106,107^ Increasing evidence in the past several years has shown that SnCs accumulate in mouse and human osteoarthritic joint tissue, and SASP has been implicated in osteoarthritis progression.^108–111^ SnCs typically develop in response to cellular stress, preventing the proliferation of damaged or dysfunctional cells, thereby protecting organisms against diseases. Initially, SnCs are a crucial component of the wound-healing process; however, if they are not cleared in a timely manner, their persistence can lead to tissue deterioration that causes aging, as well as many chronic conditions and age-related diseases.^56,58,112^ Senescent chondrocytes, for example, have been linked to post-traumatic osteoarthritis (PTOA) and age-related osteoarthritis since they were identified in the cartilage tissue isolated from patients with osteoarthritis undergoing joint replacement surgery.^11,108,113^

As osteoarthritis progresses, the chondrocyte phenotype is skewed toward matrix degradation accompanied by SnC-associated SASP factors, specifically matrix metalloproteinases (MMP)-13 and aggrecan-degrading ADAMTS-5.^114,115^ In addition, oxidative stress promotes chondrocyte senescence, mainly by activating p38 MAPK and PI3K/Akt signaling, leading to SASP factor production and increased inflammation and tissue deterioration.^116^ Macrophages, in response to the SASP, are recruited to the articular space, secrete proinflammatory factors interleukin (IL)-1β, IL-6, and tumor necrosis factor α (TNFα), as well as growth factors TGFβ and BMPs, which activate synovial fibroblasts to upregulate MMPs and ADAMTS and promote osteophyte formation and drive osteoarthritis progression.^117–119^ More recently, we have shown that damage to the joint (anterior cruciate ligament transection [ACLT]) results in increased cellular senescence (p16^+^), upregulated SnC-associated proinflammatory factors, as well as a Th17-type immune signature in the joint and draining lymph nodes, and ultimately osteoarthritis development.^108,110^ Historically, osteoarthritis has been defined as a primarily localized disease, but through in vitro and in vivo studies we have identified a systemic Th17-SnC feedback where Th17 T cells induce senescence in healthy fibroblasts and SnCs skew naive T cells toward a Th17 phenotype in the presence of TGFβ.^110^ Thus, with this new understanding of the systemic immunological feedback that exacerbates cartilage degeneration leading to osteoarthritis, more effective therapeutics can be developed to combat not only the pathological SnC, but also the greater effects of their SASP. The immune system initiates and conducts critical responses to tissue damage. SnCs, through their SASP, secrete several cytokines that influence the immune response orchestrating the balance between a proinflammatory and pro-regenerative response to injury or trauma.^56,112,120^ Trauma induces a cascade of local and systemic immune events that recruit various cells to the injured site to protect the tissue from infection and promote tissue repair. Cartilage, however, because of its avascular composition, has limited self-repair capacity. Therefore, SnC accumulation and SASP signaling leading to chronic inflammation in the joint after trauma not only results in the inability for cartilage repair and osteoarthritis development but also induces systemic immune changes, possibly exacerbating responses to other injuries or diseases and promoting further tissue deterioration. The canonical proinflammatory molecules associated with SnC accumulation and their SASP in osteoarthritis are cytokines (IL-1β, IL-6, TNFα), chemokines (CCL2, CCL4), proteases (MMPs, ADAMTS), and growth factors (TGFβ, IGFBP).^109^ The identification of the SnCs in the osteoarthritic joints and their contribution to the disease development have also been discussed in recent comprehensive review articles.^109,121^

Osteoarthritis has heterogeneous causes that lead to accelerated structural damage and has long been considered as the unique consequence of a “wear and tear” process leading to cartilage degradation. Indeed, direct joint injury and mechanical overloading are important contributors to the development of osteoarthritis.^122,123^ However, this initial paradigm has been modified during the past decade because of critical findings from epidemiologic and prospective clinical studies. Specifically, only 12% of the overall prevalence of symptomatic osteoarthritis is attributable to PTOA of the hip, knee, or ankle,^124^ indicating that mechanisms other than biomechanical factors are involved in osteoarthritis development. Older age is the greatest risk factor for osteoarthritis, and age-related changes could accelerate the development of its pathologic process. Furthermore, metabolic osteoarthritis has now been considered as a subtype of osteoarthritis defined by the presence of individual metabolic syndrome (MetS) components or MetS as a whole.^125^ Epidemiologic studies have shown that osteoarthritis is strongly associated with cardiovascular disease,^126–129^ and the rate of cardiovascular mortality is directly proportional to the extent of radiographic evidence of osteoarthritis. Furthermore, osteoarthritis patients have a higher prevalence of cardiovascular risk factors, including dyslipidemia, obesity, hypertension, and DM,^130–133^ all of which are aspects of MetS. Particularly, 59% of patients with osteoarthritis had MetS compared with 23% of the general population, and the middle-aged population with osteoarthritis has more than a five-fold increase in the risk of MetS compared with the population without osteoarthritis.^134^ Although aging remains the most important risk factor for osteoarthritis, the metabolic phenotype has become the second most frequent subtype of osteoarthritis among patients enrolled in clinical studies.^125,135–137^ However, the pathogenic mechanisms of osteoarthritis associated with MetS remain unclear. It will be important to know whether cellular senescence is also involved in the pathogenesis of MetS-associated osteoarthritis. Understanding the role of cellular senescence and the SASP in the development of different subtypes of osteoarthritis will undoubtedly inform the development of targeted treatment approaches to stop or slow osteoarthritis progression.

### Seno-therapy to treat osteoarthritis

Despite of the intensive investigations in the past two decades on the pathogenesis of osteoarthritis, currently there are still no FDA-approved medications to slow or stop the disease progression. Cellular senescence and the SASP have causal roles in mediating many age-associated chronic diseases. Therapeutically directly eliminating SnCs or targeting the effects of SnCs hold promise to prevent, delay, or alleviate these conditions. To address the recent findings of the effect of SnC and the SASP in osteoarthritis, researchers have been investigating the efficacy of two families of senescent-related medications: senolytics and senomorphics. Senolytics preferentially induce apoptosis in SnCs. Senomorphics, also referred to as senostatics or SASP inhibitors, selectively block SASP factors that cause further inflammatory paracrine signaling and tissue damage.^138^

**Fig. 4:**
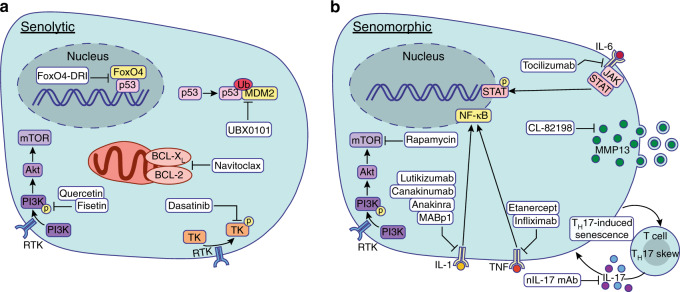
Senolytic and senomorphic therapeutic strategies. **a** Senolytic strategies seek to preferentially induce apoptosis in senescent cells and target molecules or signaling pathways that are increased in senescent cells such as p53, BCL-X_L_ and BCL-2, and survival signaling through receptor tyrosine kinases (RTKs). **b** Senomorphic strategies target SASP factors that can cause further inflammatory paracrine signaling and tissue damage. CL82198, a selective MMP13 inhibitor, and rapamycin, an mTOR inhibitor, are both senomorphics under assessment. Blocking antibodies as well as neutralizing antibodies for several SASP factors (IL-1, IL-6, TNF) are being tested for efficacy against further tissue damage. Neutralizing IL-17a/f monoclonal antibodies (nIL-17 mAb) are under evaluation to disrupt the feedforward cycle of T_H_17 skewing and T_H_17-induced senescence. Ub Ubiquitin

In initial studies exploring the role of senescence in age-associated abnormalities, mice that had undergone clearance of p16-expressing cells had an increased lifespan and delayed onset of age-related diseases.^139,140^ When a similar inducible p16-clearance model was used in osteoarthritic mice, the development of osteoarthritis was attenuated and cartilage repair and development increased.^108^ Senolytics typically induce preferential or ideally selective apoptosis of SnCs via targeting pro-survival signals and pathways that are activated in SnCs but not healthy cells (Fig. 4a). Timing of delivery, however, is critical with senolytic therapies because SnCs are crucial to the initial tissue injury response; therefore, senolytics are typically administered after the initial inflammatory phase, when tissue repair must be promoted. Navitoclax, a BCL-2 and BCL-Xl inhibitor, clears senescent, p16^+^ stem cells in the bone marrow and muscle, thus promoting regeneration when delivered systemically.^141–143^ Other pro-survival targets constitute several tyrosine kinases, such as BCR-ABL, SRC, c-KIT, ephrin A receptor, and PDGF-β-receptor tyrosine kinases. Dasatinib is a senolytic drug that targets all of the aforementioned tyrosine kinase targets and has been used in combination with quercetin, which inhibits PI3K and serpins.^144^ The dual treatment of dasatinib and quercetin eliminates SnCs in the cartilage and bone and promotes tissue regeneration.^97,145,146^

UBX0101, via local intra-articular injection, is a senolytic that is currently being assessed in clinical trials for its efficacy in osteoarthritis treatment (ClinicalTrials.gov identifiers NCT03513016, NCT04229225, and NCT04349956). UBX0101 inhibits p53 ubiquitination and degradation by preventing MDM2, an E3 ubiquitin protein ligase, binding and has shown promise by eliminating SnCs and relieving osteoarthritis-related articular cartilage degradation and proteoglycan loss via local delivery.^108^ Another study sought to determine whether local and/or systemic senolytic delivery promotes cartilage repair.^110^ ACLT was performed in young and aged mice to create a model of PTOA. Mice were then treated with local UBX0101, systemic navitoclax, or both. Young mice responded well to solo treatment with either UBX0101 or navitoclax, showing improved tissue structure, decreased osteophytes, and potential tissue repair, whereas aged mice experienced significant improvement only with both local and systemic delivery.^110^ Faust et al.^110^ concluded that systemic senolysis is required in aged animals to restore a wound-healing and tissue-regeneration capacity. Furthermore, sirtuin-1 (SIRT1) is a crucial enzyme to ECM homeostasis and chondrocyte survival,^147^ and SIRT1 cleavage has been correlated with chondrosenescence and osteoarthritis.^148^ In another study, ACLT in mice induced SIRT1 cleavage, and systemic navitoclax delivery combined with intra-articular UBX0101 delivery decreased the amount of circulating cleaved SIRT1.^148^ Another senolytic that is currently being assessed in clinical trials is fisetin (NCT04210986). Fisetin, a flavonoid that activates sirtuins such as SIRT1, has been suggested to increase longevity and inhibit IL-1β–propagated inflammation in osteoarthritis.^149,150^ Via high-throughput drug screening against senescence in chondrocyte cell lines, new senolytics can be identified for further investigation.

Senomorphic strategies target SASP factors that can influence the surrounding tissue microenvironment by inflammatory paracrine signaling (Fig. 4b). SASP factors fall into two main classes: MMPs and cytokines. Of MMPs, the most highly expressed is MMP13, which can efficiently degrade type-II collagen in articular cartilage.^151^ Patients with osteoarthritis express higher levels of MMP13 in their chondrocytes than do healthy patients,^152^ and MMP13 has been identified as a primary driver of osteoarthritis.^153^ CL82198, an MMP13 inhibitor, decreased osteoarthritis-related degeneration and pain, decreased chondrocyte cell death, and increased type-II collagen levels.^154^ Therefore, MMP13 inhibitors could be a promising therapy for osteoarthritis patients.

TNF and IL-1β are proinflammatory cytokines that promote MMP production and secretion, preventing tissue regeneration and repair. Unfortunately, targeting these two cytokines has produced little clinical improvement in osteoarthritis trials, with little or no effects on pain and tissue repair.^155,156^ A more recent study, however, indicated a lower incidence of hip and knee replacement in a group treated with anti-IL-1β antibody compared with the placebo group.^157^ Clinically approved for treatment of rheumatoid arthritis, an anti-IL-6-receptor, tocilizumab, is being assessed for clinical efficacy for osteoarthritis of the hand (NCT02477059). One study reported that IL-6 knockout mice experience more severe osteoarthritis than wild-type mice^158^ so targeting certain immunologic mediators involved in the SASP is not straightforward. More recently, however, IL-17 was linked to the SASP profile, causing inflammation-induced SnCs.^110,142^ In aged animals, co-administration of local and systemic senolytics attenuated tissue degeneration after ACLT and corresponded to decreased IL-17 expression in the joint and draining lymph nodes.^110^ In vitro, SnCs skewed naive T cells toward a proinflammatory Th17 or Th1 type, while Th17 T cells were also able to induce senescence in fibroblasts, indicating a feedback loop that perpetuates senescence development and tissue damage.^110^ Faust et al.^110^ assessed the efficacy of an IL-17 neutralizing antibody against further osteoarthritic damage after ACLT and found that intra-articular injection of an anti-IL-17a neutralizing antibody decreased MMP13 and p21 expression, significantly improved tissue structure, and relieved SnC burden and the pathologic effect of cartilage injury. Therefore, future studies should explore the connection between IL-17 and senescence and the efficacy of IL-17-blocking senomorphics to promote cartilage regeneration.

## Cellular senescence in the pathogenesis of sarcopenia

### Cellular senescence in sarcopenia

Skeletal muscle attaches to bone via strong connective tissues and therefore is key to controlling locomotion. Skeletal muscle is also a critical factor in whole-body metabolism and energy expenditure and is essential for maintaining quality of life.^159–162^ Skeletal muscle contains several cell populations, including myocytes, skeletal stem cells, fibroblasts, nerve fibers, and blood vessel cells. Adult muscle stem cells are referred to as satellite cells, which are responsible for muscle maintenance, repair, and regeneration in aging and injured muscles.^160,163^ Loss of lean muscle mass begins at a slow rate around the fifth decade of life and accelerates over time, resulting in 30%–50% of lean mass reduction by age 70.^164,165^ Muscle strength deficiency precedes muscle loss and declines much faster than muscle mass. Age-related decline in muscle mass and associated muscle weakness are referred to as sarcopenia.^166^

Preliminary studies have linked increased senescence, not only of the muscle cells themselves, but also of their SASP, to the characteristic muscle-fiber thinning of sarcopenia. A recent study sought to determine whether SnC-implanted adjacent to skeletal muscle would affect healthy surrounding muscle cells via the “bystander effect” and senescent-like signaling.^167^ SnC transplantation adjacent to skeletal muscle induced an increase in senescent markers in the muscle, as well as muscle-fiber thinning, indicative of sarcopenia.^167^ SASP factors have also been linked to the induction of several factors, such as sarcolipin, that have been shown to promote skeletal muscle fibrosis and, ultimately, sarcopenia.^168^

Other factors, such as endothelin-1, TRIM32, and GSK-3α have been shown to induce muscle stem cell senescence and suppress autophagy in mouse models. Knockout of each of these factors results in muscle degeneration and sarcopenia development.^169–171^ In addition, aberrant p38α/β ΜΑPK signaling has also been observed in aged muscle stem cells, leading to reduced regenerative capacity and functional decline.^172^ Pharmacologic inhibition of this p38α/β signaling in preclinical studies enhances aged muscle stem cell proliferation, restores aged muscle stem cell regenerative capacity, and provides long-term functionality and increased strength. Furthermore, in murine muscle stem cells, a recent study reported reduced expression of the transcription factor *Slug* and identified *Slug* as a transcriptional repressor of senescence-initiating gene p16^Ink4a^.^173^ The authors proposed to develop therapeutics to overexpress *Slug* as a potential inhibitor of age-related skeletal muscle degeneration and even sarcopenia development. Wnt-3 is another therapeutic target against skeletal muscle senescence and deterioration. Wnt-3 was increased in the serum of elderly people, and Wnt-3 expression induces CCN1 expression.^174^ Any of these molecules could serve as promising potential targets for combatting age-related muscle degeneration and promote muscle regeneration in cases of sarcopenic development.

Autophagy is the organized degradation and recycling of dysfunctional cellular components. Typically, in muscle stem cells, autophagy activity declines with age, resulting in accumulation of damaged cell components and a transition from quiescence to senescence.^175^ Maintenance of basal autophagy in muscle stem cells is critical for preventing senescence and maintaining regenerative capacity.^176^ Therefore, promotion of basal autophagy in aged muscle is a promising therapy to reverse senescence in muscle stem cells in elderly populations.^175^ Some disorders in which autophagy is impaired lead to sarcopenia via senescence induction. For example, hyperphosphatemia, an electrolyte disorder in which circulating phosphate levels are higher than normal, induces myoblast senescence via autophagy impairment and integrin-linked kinase overexpression, which could propagate muscle degeneration and sarcopenia.^177^

Recent studies have begun investigating immunogenic changes that can be influenced by SnCs in the development of sarcopenia. Preliminary studies have shown that bone marrow transplants from young mice into old mice prevent sarcopenia.^178,179^ Macrophages present in muscle repair have been shown to secrete nicotinamide phosphoribosyltransferase (NAMPT) to stimulate muscle stem cells and promote myoblast proliferation.^180^ Because NAMPT has been shown to inhibit senescence in endothelial progenitor cells via SIRT1 regulation,^181^ the study suggests that macrophages, by producing NAMPT, may prevent/inhibit cellular senescence and the corresponding SASP and therefore positively regulate muscle regeneration.

Metabolic irregularities, which disrupt typical metabolic homeostasis, can lead to systemic inflammation and senescence. Aberrant metabolic signaling in skeletal muscle, a critical metabolic tissue, contributes to senescence and the SASP, which accelerates sarcopenia.^182,183^ Oxidative stress induces senescence in mesenchymal progenitor cells, which typically support myogenic cell function but can further differentiate into fibrous/adipose tissue that is detrimental to skeletal muscle support.^184^ These senescent mesenchymal progenitor cells, when cultured with myoblasts, prevented myotube formation and accelerated sarcopenia.^185,186^ Several metabolic enzymes are involved in metabolic sarcopenia. Peroxiredoxin 6, an antioxidant enzyme, prevents SIRT1 cleavage, as well as forkhead box O1 (FoxO1) expression, and when absent leads to muscle degeneration associated with sarcopenia.^187^ Increased FoxO expression increases proteolysis, leads to increased senescence, and accelerates age- and obesity-induced muscle degeneration.^188^ The role of perimuscular adipose tissue accumulation around muscle has also been explored in the development or sarcopenia. Perimuscular adipose tissue transplantation in mice increased activation and nuclear translocation of FoxO transcription factors.^188^ Optic atrophy 1 (OPA1), another general metabolic regulator, is a mitochondrial protein in which decreased levels have been associated with sedentary but not active people experiencing muscle loss.^189^ Via FGF21, OPA1 regulates muscle stem cell activity and senescence, and OPA1 abrogation leads to endoplasmic reticulum stress and induction of FoxO transcription factors, leading to muscle catabolism and loss.^189^

These studies indicate that senescence plays a pivotal role in sarcopenia development, and a deeper understanding of senescence induction, the SASP, and sarcopenia progression would provide insight into regenerative therapeutics for muscle loss.

Senolytic and senomorphic treatments have also been used against SnCs and the SASP that contribute to sarcopenia.^190^ FoxO4 expression, a senolytic target, is elevated in SnCs and has been identified as a key molecule in maintaining SnC viability.^191^ A small molecule peptide senolytic, FoxO4-DRI, blocks FoxO4-p53 interaction, shows the ability to preferentially target SnCs, and should be assessed for efficacy against sarcopenic muscle loss.^191^ In addition, MABp1 administration, a senomorphic, efficiently targets and prevents sarcopenia progression by neutralizing anti-human IL-1α.^192^ Chung et al.^142^ explored the influence of IL-17-regulated senescence in the foreign body response after volumetric muscle loss and found that SnC development was abrogated in IL-17A^–/–^ and IL-17RA^–/–^ mice, indicating that SnC induction is dependent on IL-17. This finding could indicate that a senomorphic IL-17-blocking therapeutic, as discussed in the cartilage repair section, could be a promising strategy to attenuate further senescence/SASP–related damage and promote muscle repair and regeneration. In addition, treatment with an IL-6 neutralizing antibody showed decreased senescent-induced *Il17a* expression, indicating that IL-6-targeted senomorphic therapies may also slow or even prevent sarcopenia progression.^142^ Finally, when navitoclax was systemically administered to mice 4 weeks after volumetric muscle loss, it cleared SnCs, reduced the SASP (particularly IL-17-related), and prevented further tissue damage.^142^ This study highlighted the efficacy of combination therapies of senolytics and senomorphics (navitoclax + anti-IL-17a/f neutralizing antibodies), illustrating that, when these treatments are combined, the SASP is even more diminished and less tissue damage occurs.^142^ These combination therapies should be assessed in the context of tissue regeneration, not only in the muscle, but also in age-related/senescence-associated bone and cartilage abnormalities.

Other treatments seek to target the factors that induce senescence. Tocotrienol-rich fraction treatment of patients with sarcopenia has been shown to reverse myoblast aging and senescence, providing muscle cells with the regenerative capacity needed to reverse muscle loss and promote repair.^193–195^ More recently, researchers have targeted microRNAs that are related to key factors that induce satellite cell senescence, such as Igfbp5, Wnt-3α signaling, and dysregulation of AMPK/SIRT1.^196–198^ Although these microRNA-targeted therapies have shown promise in preclinical trials, further studies and characterization are needed to assess the efficacy in preventing pathological senescence, reversing sarcopenic muscle loss, and promoting regeneration.

## Summary and perspective

With aging, various mechanistic changes occur in the MSK, which result in structural degeneration, mechanical pain, decreased mobility, and limited function. The degeneration of bones, joints, and skeletal muscles accelerates frailty and makes older people prone to osteoporosis, osteoarthritis, and sarcopenia. During the past two decades, studies using mouse models have established that SnCs accumulate in different components of the MSK with aging, and elimination of SnCs results in attenuation/deceleration of disease progression. However, several issues need to be resolved before interventions to remove SnCs are tested in humans to treat age-associated disorders in the MSK. First, it is important to understand the spatio-temporal distribution of SnCs in human musculoskeletal tissues, under diverse conditions and during all stages of the lifespan. Second, we must explore the heterogeneity of SASP in different cell types in the complex MSK microenvironment. Third, although excessive accumulation of SnCs is associated with increased susceptibility to chronic musculoskeletal diseases, SnCs also have beneficial physiologic functions during embryonic development, late pubertal bone growth cessation, and tissue remodeling in adulthood. Therefore, it is imperative to better understand the mechanisms that distinguish beneficial from deleterious senescence and the heterogeneity underlying SnC states. Mapping the landscape of cellular senescence under physiologic or pathologic conditions will undoubtedly aid the targeted development of therapeutic approaches for SnCs. Transformational advances in the combined use of single-cell technologies and in situ image-based approaches would allow us to generate a molecular atlas of SnC types in vivo while mapping their spatial and functional organization.
